# Relationship between Glycated Hemoglobin in Adolescents with Type 1 Diabetes Mellitus (T1DM) and Parental Anxiety and Depression

**DOI:** 10.1192/j.eurpsy.2023.1430

**Published:** 2023-07-19

**Authors:** E. Silina, M. Taube, M. Zolovs

**Affiliations:** 1 Doctoral studies, Riga Stradins University, Riga; 2 The Seaside Hospital, Liepaja; 3 Department of Psychiatry and Narcology; 4 Statistics Unit, Riga Stradins University, Riga; 5 Institute of Life Sciences and Technology, Daugavpils University, Daugavpils, Latvia

## Abstract

**Introduction:**

T1D is the most common chronic endocrine pathology in children. The management of type 1 diabetes requires strong diet, physical activity, lifelong insulin therapy, and proper self-monitoring of blood glucose and is usually complicated and, therefore may result in a psychosocial problems for the whole family. Metabolic control of the disease is determined by glycated haemoglobin (HbA1c), the main criterion for diabetes compensation. It is assumed that anxiety and depression symptoms negatively affect glycaemic control. Parental psychological distress was associated with higher child self-report of stress and depressive symptoms, and it had negative effects on diabetes management.

Type 1 diabetes mellitus (T1D) is the most common chronic endocrine pathology in children. The management of type 1 diabetes requires strong diet, physical activity, lifelong insulin therapy, and proper self-monitoring of blood glucose and is usually complicated and, therefore may result in a variety of psychosocial problems for children, adolescents, and their families. Metabolic control of the disease is determined by glycated haemoglobin (HbA1c), the main criterion for diabetes compensation. A correlation is observed between anxiety and depression level and glycaemic control in many previous studies. It is assumed that anxiety and depression symptoms negatively affect glycaemic control. Parental psychological distress was associated with higher child self-report of stress and depressive symptoms, and it had negative effects on diabetes management.

**Objectives:**

To evaluate the relationship between parental depression and anxiety and metabolic control of their adolescents with T1DM.

**Methods:**

Cross-sectional study recruited adolescents with T1D (N=251) and their parents (N=251). Anxiety level was measured by 7-item Generalized Anxiety Disorder (GAD-7) scale. Depressive symptms wasdetected using The Patient Health Questionnaire – 9 (PHQ-9). Glycaemic control of patients was assessed using the last HbA1c values. GLM mediation analysis was performed to determine the potential mediating effect of parental depression and anxiety on the relationship between depression and anxiety of the child on the level of glycated hemoglobin.

**Results:**

502 respondents were eligible for screening. Mediation analysis was performed to assess the mediating role of parent GAD-7 on the linkage between HbA1c, child GAD-7 and child PHQ-9. The total effect of child GAD-7 on HbA1c was significant but the total effect of child PHQ-9 was not. With the inclusion of the mediating variable (parent GAD-7) (Figure 1), the indirect effect of child GAD-7 and the child PHQ-9 on HbA1c though parent GAD-7 was found significant (Table 1).

**Image:**

**Figure:**
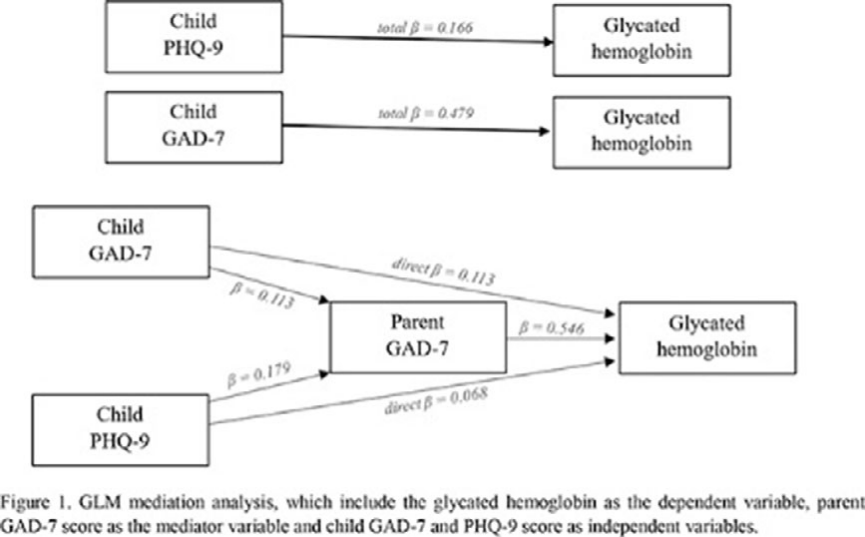

**Image 2:**

**Figure:**
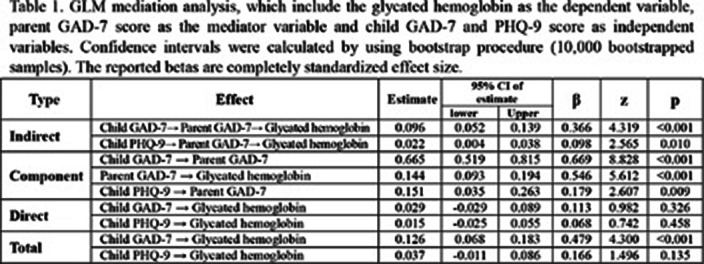

**Conclusions:**

Parental anxiety is a significant risk factor for child depression and anxiety, which determines poorer T1D metabolic compensation ard worse HbA1C scores.

**Disclosure of Interest:**

None Declared

